# Dual-Energy CT Pulmonary Angiography for the Assessment of Surgical Accessibility in Patients with Chronic Thromboembolic Pulmonary Hypertension

**DOI:** 10.3390/diagnostics12020228

**Published:** 2022-01-18

**Authors:** Matthias Eberhard, Micheal McInnis, Marc de Perrot, Mona Lichtblau, Silvia Ulrich, Ilhan Inci, Isabelle Opitz, Thomas Frauenfelder

**Affiliations:** 1 Institute of Diagnostic and Interventional Radiology, University Hospital Zurich, 8091 Zurich, Switzerland; thomas.frauenfelder@usz.ch; 2 Radiology, Spitäler fmi AG, 3800 Interlaken, Switzerland; 3 Joint Department of Medical Imaging, University of Toronto, Toronto, ON M5T 1W5, Canada; micheal.mcinnis@uhn.ca; 4 Division of Thoracic Surgery, Princess Margaret Cancer Centre (Toronto General Hospital), University Health Network, Toronto, ON M5G 2A2, Canada; marc.deperrot@uhn.ca; 5 Department of Pulmonology Zurich, University Hospital Zurich, 8091 Zurich, Switzerland; mona.lichtblau@usz.ch (M.L.); silvia.ulrich@usz.ch (S.U.); 6 Department of Thoracic Surgery, University Hospital Zurich, 8091 Zurich, Switzerland; ilhan.inci@usz.ch (I.I.); isabelle.schmitt-opitz@usz.ch (I.O.)

**Keywords:** computed tomography, hemodynamics, pulmonary artery, pulmonary hypertension

## Abstract

We assessed the value of dual-energy CT pulmonary angiography (CTPA) for classification of the level of disease in chronic thromboembolic pulmonary hypertension (CTEPH) patients compared to the surgical Jamieson classification and prediction of hemodynamic changes after pulmonary endarterectomy. Forty-three CTEPH patients (mean age, 57 ± 16 years; 18 females) undergoing CTPA prior to surgery were retrospectively included. “Proximal” and “distal disease” were defined as L1 and 2a (main and lobar pulmonary artery [PA]) and L2b-4 (lower lobe basal trunk to subsegmental PA), respectively. Three radiologists had a moderate interobserver agreement for the radiological classification of disease (k = 0.55). Sensitivity was 92–100% and specificity was 24–53% to predict proximal disease according to the Jamieson classification. A median of 9 segments/patient had CTPA perfusion defects (range, 2–18 segments). L1 disease had a greater decrease in the mean pulmonary artery pressure (*p* = 0.029) and pulmonary vascular resistance (*p* = 0.011) after surgery compared to patients with L2a to L3 disease. The extent of perfusion defects was not associated with the level of disease or hemodynamic changes after surgery (*p* > 0.05 for all). CTPA is highly sensitive for predicting the level of disease in CTEPH patients with a moderate interobserver agreement. The radiological level of disease is associated with hemodynamic improvement after surgery.

## 1. Introduction

Chronic thromboembolic pulmonary hypertension (CTEPH) is a rare, progressive pulmonary vascular disease [1,2]. It is most commonly a sequela of prior venous thromboembolism with an incidence between 0.4% and 6.2%, confirmed by right heart catheterization [2,3,4]. However, due to the unspecific symptoms, the precise incidence remains unknown.

Pulmonary endarterectomy is the treatment of choice for CTEPH and three-year survival in those patients undergoing pulmonary endarterectomy (90% three-year survival) is significantly improved compared to those who are not operated on (70% three-year survival) [5,6,7]. Surgical accessibility is determined in part by the most proximal extent of chronic thromboembolic changes [8,9]. However, this Jamieson classification scheme is based on intraoperative findings and there are limited reports on its correlation with preoperative imaging [9,10]. Patients deemed inoperable based on comorbidities, or with extensive distal disease not accessible to surgery, may be candidates for medical therapy and/or balloon pulmonary angioplasty but these therapies are not curative [1,11].

Computed tomography (CT) has excellent spatial and temporal resolution, enabling a comprehensive evaluation of cardiopulmonary structures within a single breath hold [12,13,14]. Contrast-enhanced CT pulmonary angiography (CTPA) allows for the non-invasive assessment of chronic thromboembolic changes in the pulmonary arteries [15]. Using state-of-the-art equipment with a 64-row to 320-row multidetector CT or dual-energy CT, CT pulmonary angiography is as accurate as ventilation-perfusion scintigraphy for the detection of CTEPH [16,17]. Moreover, CTPA allows for a non-invasive assessment of surgical accessibility as an alternative to invasive digital subtraction angiography [18,19]. McInnis et al. demonstrated that the preoperative CTPA strongly correlates with surgical findings [20]. Dual-energy CT allows the creation of iodine maps providing an assessment of lung perfusion but the role of this additional test in the preoperative assessment is unclear.

This study aimed (i) to assess the predictive value of a radiological classification of the level of disease in CTEPH patients on CTPA using the surgical classification as a standard of reference, (ii) to assess whether perfusion defects assessed on dual-energy CT iodine maps correlate with the radiological level of disease assessed on CTPA, and (iii) to assess the association of the radiological level of CTEPH and the extent of perfusion defects with the improvement of hemodynamics after surgery.

## 2. Methods

Patient Population:

The institutional review board approved this retrospective study. Written informed consent was obtained from all patients.

We retrospectively screened 64 patients who underwent surgical pulmonary endarterectomy for treatment of CTEPH between May 2015 and January 2020. Exclusion criteria were preoperative CTPA not performed with dual-energy CT in-house (*n* = 18), respiratory motion artifacts (*n* = 2), and mistimed opacification of the pulmonary arteries (*n* = 1). In all patients included in the final analysis (*n* = 43), the diagnosis of CTEPH was confirmed in a multidisciplinary conference, including a thoracic surgeon, a pulmonologist, and a radiologist following guidelines for the diagnosis of group 4 pulmonary hypertension [21].

### 2.1. CTPA Acquisition and Image Reconstruction

All patients underwent single or dual-energy CTPA on a third-generation dual-source CT scanner (SOMATOM Force; Siemens Healthineers, Forchheim, Germany).

Dual-energy CT was acquired in mid-inspiratory breath-hold with the following parameters: tube voltage, 90 kVp (tube A) and 150Sn kVp (tube B). Automatic tube current, reference tube current (effective mAs) was 110 (tube A)/85 (tube B). A biphasic contrast media injection protocol using 60 mL of contrast media (iopromide, 300 mg iodine/mL; Ultravist300, Bayer Pharma AG, Berlin, Germany) was injected into an antecubital vein: in phase I, 40 mL of contrast media (concentration 100%), and in phase II, 20 mL of contrast media diluted with 20 mL of saline solution (NaCl, 0.9%) was administered. Flow rates were 3.5 mL/s in phase I and 2.5 mL/s in phase II.

All images were reconstructed with a section thickness of 2 mm and an increment of 1.6 mm, Advanced Modeled Iterative Reconstruction (ADMIRE) at strength level 3, and a mediastinal soft-tissue convolution kernel (Bf40). Axial and coronal dual-energy iodine maps were generated using the Lung Analysis application of the Syngo.via workstation (Siemens Healthineers).

### 2.2. Data Collection

Baseline clinical history was systematically collected at the time of surgery. Data from the 6 min walking test and echocardiography were collected at baseline and 3 months after surgery. Data from right heart catheterization were collected at baseline and 12 months after surgery. The surgical level of disease was extracted from the operative report.

### 2.3. Analysis of Pulmonary Arteries

According to the surgical Jamieson classification (afterwards, simply known as Jamieson classification), the level of disease was judged according to the most proximal chronic thromboembolic changes: level I, involvement of the main pulmonary arteries; level II, involvement of lobar arteries; level III, involvement of the segmental arteries and level IV, involvement of subsegmental arteries predominantly [9].

Three radiologists blinded to surgical results (M.M., 4 years of experience in thoracic radiology; T.F., 15 years of experience in thoracic radiology and M.E., 6 years of experience in thoracic radiology) reviewed CT images using a commercially available picture archive and communication system (Impax 6.5.5; Agfa HealthCare, Mortsel, Belgium). Multiplanar reformations of axial images were applied to assess changes due to chronic thromboembolism, including total or partial occlusion, eccentric thrombus (with or without calcification), or slits and webs.

**Figure 1:**
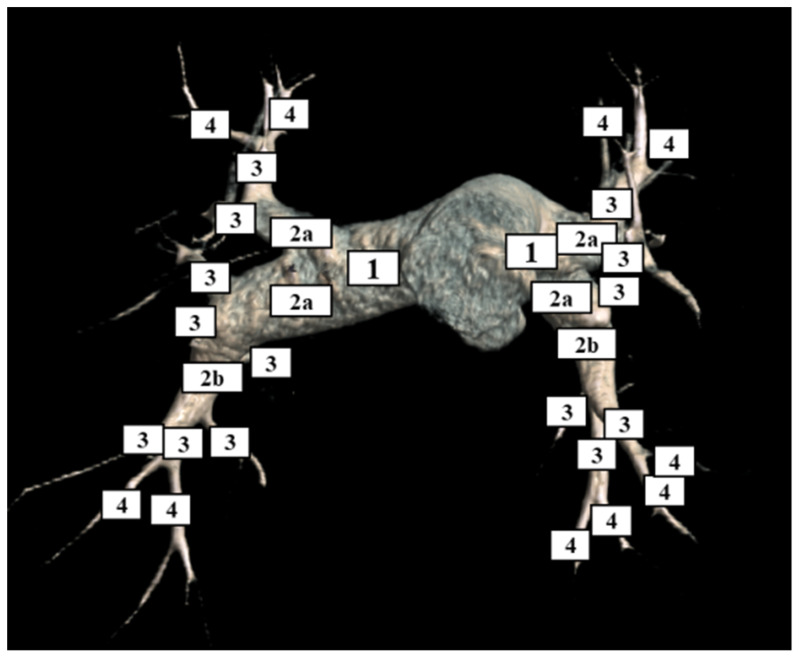
Computed tomography volume rendering of the pulmonary arteries illustrating the classification of the level of disease in patients with chronic thromboembolic pulmonary hypertension. The classification is based on the location of the most proximal thrombus. Level 1 disease was defined as any disease involving the main, left, or right main pulmonary arteries. Level 2a disease was defined as any disease in the interlobar or lobar pulmonary arteries. Level 2b disease was defined as disease in the basal trunk of the pulmonary artery of the lower lobe distal to the superior segmental vessel origin. Level 3 disease was defined as disease in the segmental pulmonary arteries within 1 cm of its origin. Level 4 disease was defined as disease more distal than 1 cm from the origin of the segmental vessel, including the subsegmental vessels.

The radiologic levels of disease were assigned based on the most proximal disease present (Figure 1), according to the Jamieson classification with the additional separation of Level 2 into Level 2a and 2b according to prior investigation [9,20]. Level 1 disease was defined as any disease involving the main, left, or right main pulmonary arteries. Level 2a disease was defined as any disease in the interlobar or lobar pulmonary arteries. Level 2b disease was defined as disease in the basal trunk of the pulmonary artery of the lower lobe distal to the superior segmental vessel origin. Level 3 disease was defined as disease in the segmental pulmonary arteries within 1 cm of its origin. Level 4 disease was defined as disease more distal than 1 cm from the origin of the segmental vessel, including the subsegmental vessels.

### 2.4. Analysis of Perfusion Defects

One reader (T.F., 15 years of experience in thoracic radiology) blinded to clinical data and to results of the analysis of pulmonary arteries analyzed perfusion defects on iodine maps using axial and coronal data sets. A second reader (M.E., 6 years of experience in thoracic radiology) performed a readout of 15 cases to assess interobserver agreement. The number of segments with perfusion defects were counted and all lung segments were rated as 0 (no perfusion defect), 1 (subsegmental perfusion defect), or 2 (segmental perfusion defect) to derive a site-specific and overall perfusion defect score (PD score) summarizing the extent of peripheral perfusion defects in each patient.

### 2.5. Statistical Analysis

Normality was assessed using the Shapiro–Wilk test. Categorical data are given as count (percentage). Continuous data are given as the mean ± standard deviation or median and inter-quartile range (IQR), where appropriate. Comparison between groups was made using the Kruskal–Wallis or the one-way ANOVA test where appropriate, both with post hoc testing. Fleiss’ kappa was applied to calculate interrater agreement of level of disease classifications. The correlation of continuous data was assessed using Pearson’s correlation coefficient. To assess the predictive value, level 1 and 2a disease were summarized as “proximal disease”, and level 2b, 3, and level 4 were summarized as “distal disease” according to McInnis et al. [20]. Interobserver agreement of continuous data was assessed using the intraclass correlation coefficient (ICC). A two-sided *p*-value of 0.05 was considered statistically significant. All analyses were performed using SPSS for Windows 25.0 (Chicago, IL, USA).

## 3. Results

### 3.1. Patient Characteristics

In total, 43 patients were included (mean age 57 ± 16 years; 18 females). All patients underwent bilateral pulmonary endarterectomy. Baseline characteristics are presented in Table 1. The median time between CTPA and surgery was 77 days (range, 1–248 days). According to surgery, the level of disease was level I in 14 patients (33%), level II in 12 patients (28%), level III in 16 patients (37%), and level IV in 1 patient (2%) of 43 patients.

### 3.2. CT Categorization of Thromboembolic Level of Disease

#### 3.2.1. Interobserver Comparison

**Figure 2:**
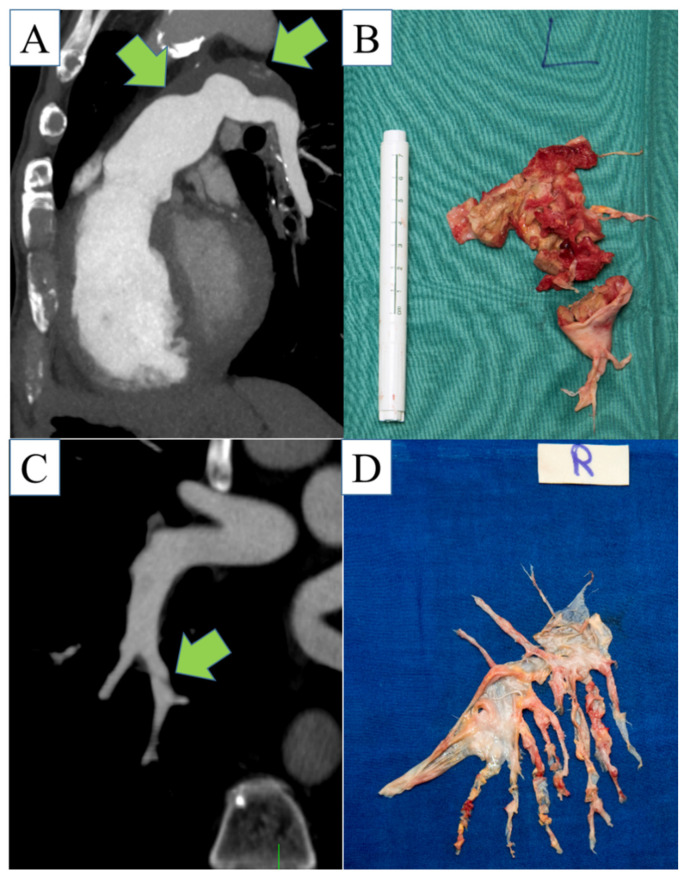
Exemplary cases: Panel (**A**) (multiplanar CT reconstruction) and (**B**) (surgical specimen) show a case of extensive chronic thromboembolic changes in a 64-year-old male patient involving the left pulmonary artery. Panel (**C**) (multiplanar CT reconstruction) and (**D**) (surgical specimen) show distal involvement chronic thromboembolic changes in a 54-year-old male patient involving the segmental pulmonary arteries of the right lower lobe.

Three radiologists classified level 1 in 15 (35%), 12 (28%), and 9 (21%) of 43 patients. Level 2a was classified in 22 (51%), 21 (49%), and 26 (60%) of 43 patients. Level 2b was classified in 3 (7%), 6 (14%), and 3 (7%) of 43 patients, and level 3 was classified in 3 (7%), 4 (9%), and 5 (12%) of 43 patients. None of the radiologists rated level 4. All individual radiological classifications were within 1 level of the surgical classification. Table 2 shows that there was a trend toward higher classifications than surgery in all three radiologists. Sensitivity was 92–100% and specificity was 24–53% to predict proximal disease. Kappa for interobserver agreement was 0.55. Figure 2 shows two examples of radiological classifications with surgical specimen correlations.

#### 3.2.2. Consensus Classification

According to the consensus classification, 12 patients (28%) were categorized as having level 1 disease, 23 patients as having level 2a disease (53%), 3 patients (7%) as having level 2b disease, and 5 of 43 patients (12%) were categorized as having level 3 disease. Compared to the surgical classification (26 patients with proximal disease), radiologists showed a trend to classify more patients as having proximal disease (35 patients with proximal disease) as shown in Table 2 with a sensitivity of 100% and a specificity of 47%. All the patients categorized as level 2b were surgically classified as level 3.

#### 3.2.3. Assessment of Dual-Energy Iodine Maps

**Figure 3:**
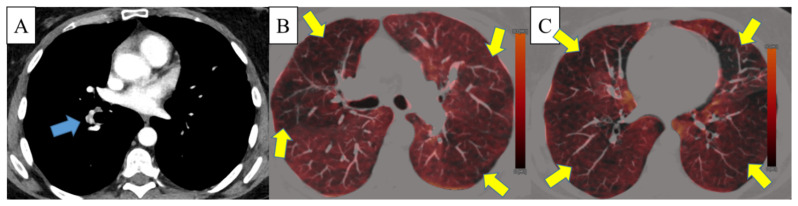
An exemplary case of a 46-year-old female with chronic thromboembolic pulmonary hypertension illustrating discrepant dual-energy CT findings of the radiological level of disease (distal disease with involvement of the basal trunk of the right lower lobe artery; level 2b, blue arrow on panel (**A**)) and extensive perfusion defects (yellow arrows on panels (**B**,**C**).

Perfusion defects were found in all CTEPH patients (*n* = 43; 100%) with a median of 9 segments (range, 2–18 segments) involved. Median PD scores of the right and left were 7 (range, 2–16) and 6 (range, 0–15), respectively. The median overall PD score was 13 (range, 3–30). Interobserver agreement for evaluation of segments with perfusion defects (ICC: 0.95; 95% CI: 0.85–0.98) as well as for the PD score (ICC: 0.95, 95%CI: 0.87–0.98) was excellent. There was no significant association of the overall number of segments with perfusion defects or PD score between radiological (*p* = 0.83 and *p* = 0.11) or surgical (*p* = 0.79 and *p* = 0.78) levels of disease in CTEPH patients (Figure 3).

### 3.3. Hemodynamic Changes after Surgery

For further comparison of disease levels and hemodynamic parameters, we report patients with chronic thromboembolism level 2b and level 3 combined as one group, due to the correlation of radiological level 2b with the surgical level III. A comparison of age, gender, and baseline hemodynamic parameters between the levels of disease classification is shown in Table 3. Patients with involvement of the main pulmonary arteries (level 1) were older (*p* = 0.018), reached a shorter distance in the six-minute walking test (*p* = 0.034), and had a higher pulmonary vascular resistance before surgery (*p* = 0.045, Table 3).

**Figure 4:**
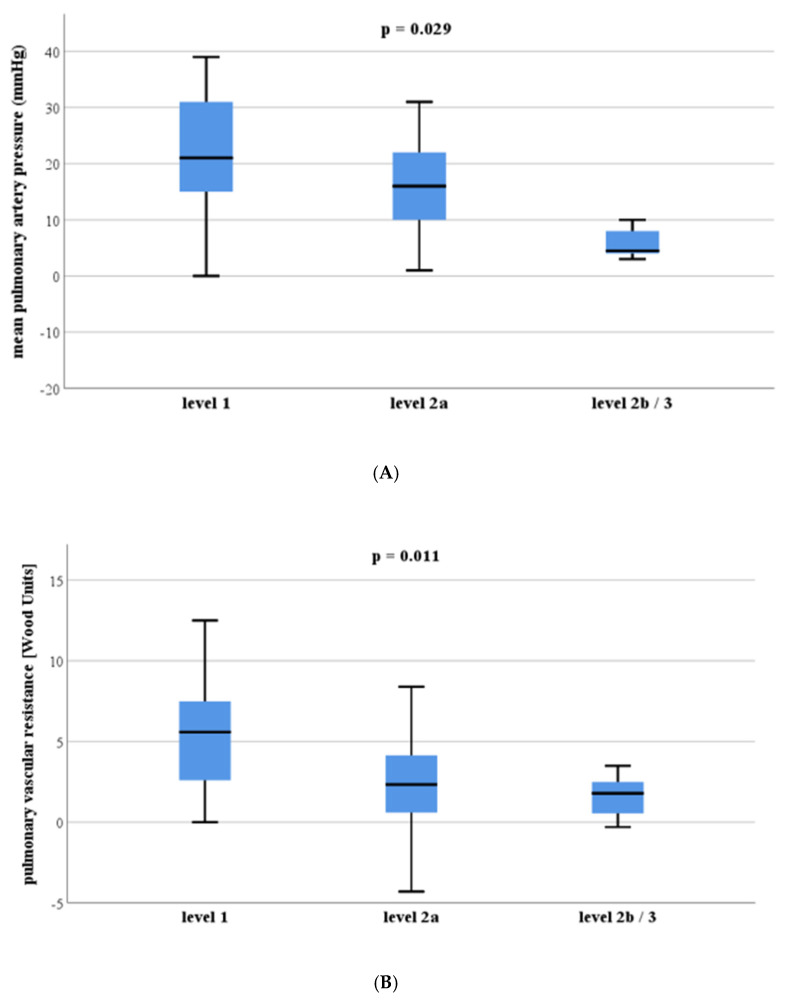
Boxplots indicate that the postoperative decrease in the mean pulmonary artery pressure (panel (**A**)) and the pulmonary vascular resistance (panel (**B**)) is significantly greater in chronic thromboembolic pulmonary hypertension (CTEPH) patients with involvement of the main pulmonary artery (level 1), compared to patients with involvement of the lobar arteries (level 2a) or involvement of the basal trunk of the lower lobe arteries or of segmental arteries (level 2b or level 3).

Changes in hemodynamic parameters according to CT categorization of the thromboembolic level of disease between preoperative baseline measurements and postoperative follow-up (3-months follow-up for walking test and echocardiography and 12-months follow-up for right heart catheterization) are shown in Table 4. CTEPH patients with involvement of the main pulmonary arteries (level 1) had a greater decrease in the mean pulmonary artery pressure (22 ± 12 mmHg) after surgery compared to patients with involvement of the basal trunk of the lower lobe arteries or of segmental arteries (level of disease 2b/3; 8 ± 6 mmHg, *p* = 0.029; Figure 4a). Furthermore, patients with involvement of the main pulmonary arteries (level 1) showed a greater decrease in pulmonary vascular resistance (5.3 ± 3.7 Wood Units, WU; Figure 4b) after surgery compared to patients with involvement of the lobar arteries (level 2a; 2.1 ± 2.8 WU, *p* = 0.018) and involvement of the basal trunk of the lower lobe arteries or of segmental arteries (level of disease 2b/3; 1.6 ± 1.4 WU, *p* = 0.036).

The number of lung segments with perfusion defects showed weak associations with the cardiac index (r = 0.35; *p* = 0.021) and pulmonary vascular resistance (r = 0.35; *p* = 0.021) at baseline. The PD score showed a weak correlation with pulmonary vascular resistance at baseline (r = 0.46; *p* = 0.002).

There was no association of the number of lung segments with perfusion defects and the PD score with the improvement of hemodynamics after surgery (*p* > 0.05 for all).

## 4. Discussion

Our data show that radiological evaluation of the level of disease in CTEPH patients classifies more patients (*n* = 35) as proximal disease than the intraoperative classification (*n* = 26) and radiological level 2b should be best classified as distal disease. Even expert radiologists only have moderate agreement assessing the radiological level of disease on CTPA (kappa 0.55 for the agreement of three readers). Involvement of the main pulmonary arteries was associated with a greater decrease in the mean pulmonary artery pressure (*p* = 0.011) and the pulmonary vascular resistance (*p* = 0.029) after surgery compared to patients with distal involvement of the pulmonary arteries. The extent of segmental lung perfusion defects was neither associated with the radiological classification of the level of disease in CTEPH patients nor was it associated with the improvement of hemodynamics after surgery (*p* > 0.05 for all).

Pulmonary endarterectomy is the treatment of choice in patients with CTEPH and all patients with CTEPH should be assessed at an expert center for eligibility [1]. However, strict objective definitions of operability remain elusive [1,9]. The selection of candidates remains subjective and is based on various factors including but not limited to the patient’s symptoms, the severity of pulmonary right heart dysfunction, and pulmonary hypertension as well as the extent and level of thromboembolic changes based on high-quality imaging [9,22]. The distal limits of endarterectomy have been redefined recently [9]; however, with inevitable variation between surgical centers [8]. Cases of CTEPH at the subsegmental level may be operable at expert centers.

Recent advancements in CT imaging have shown promising results for CTPA in the work-up of CTEPH patients. Masy et al. showed that the combination of dual-energy CT perfusion and CTPA enabled the correct classification of all patients with CTEPH in a study cohort of patients with pulmonary hypertension due to different etiologies [17]. Furthermore, for the diagnosis of CTEPH, Reichelt et al. showed that CTPA allows for the correct evaluation of thromboembolic changes with a sensitivity of 98% and a specificity of 95% at the main and lobar level and with a sensitivity of 94% and a specificity of 93% at the segmental level compared to digital subtraction angiography [18]. However, the correct classification of CTEPH changes is considered difficult and may be performed best in high volume centers due to the rarity of the disease in the general population [1,12,21]. In line with the results of McInnis et al. [20], our study showed that CTPA is highly sensitive for predicting proximal disease in CTEPH patients; however, with an overall moderate agreement with the surgical classification. Compared to the surgical classification, radiologists using CTPA images showed a trend to classify more patients as proximal disease. All patients with involvement of the basal trunk of the pulmonary artery of the lower lobe (radiological level 2b) were classified as surgical level III, confirming the results of McInnis et al. [20].

Our data show that the level of chronic thromboembolic changes in CTEPH patients is associated with the improvement of the mean pulmonary pressure and pulmonary vascular resistance 12 months after surgery. Comparable to this finding, Thistlethwaite et al. have previously shown that the surgical classification of the level of disease is associated with an improvement in hemodynamic parameters after surgery [8]. However, this association might not always hold true due to the impact of the degree of remodeling of small pulmonary vessels on hemodynamic compromise in patients with CTEPH [23].

CTPA and V/Q scintigraphy have a similar diagnostic performance for the detection of segmental pulmonary artery involvement in CTEPH [24]. Dual-energy CTPA iodine maps for the assessment of lung perfusion showed excellent agreement with V/Q scintigraphy in diagnosing CTEPH (kappa 0.8) [17]. Masy et al. showed that the combination of morphological assessment of CTPA and the evaluation of CT lung perfusion enables to correctly identify 100% of cases with CTEPH [17]. The advantage of dual-energy iodine maps may be that the evaluation of distal disease through the visibility of subsegmental perfusion defects as well as the evaluation of the extent of distal disease is improved. Tsutsumi et al. [25] previously reported a weak to moderate association of quantitative dual-energy CT parameters with hemodynamics such as the cardiac index (r = 0.49) and pulmonary vascular resistance (r = 0.48). Our data show similar results with a weak association of the extent of perfusion defects detected on dual-energy CT iodine maps with the cardiac index and pulmonary vascular resistance. However, we could not detect a significant association of the extent of perfusion defect with hemodynamic improvement in CTEPH patients after surgery. These results suggest that the addition of CT evaluation of lung perfusion to the morphological CTPA assessment may be more important for making a correct diagnosis than to predict surgical outcomes. However, larger studies may be warranted to assess this issue.

## 5. Limitations

Several limitations of our study merit consideration. First, our results were limited by the single-center observational design of the study. Second, we only included patients who underwent pulmonary endarterectomy leading to a selection bias regarding the comparison of radiological and surgical levels of disease in CTEPH patients. Third, due to the limited number of patients included, especially of patients with peripheral disease, further studies should assess the reproducibility of our results. Fourth, women were underrepresented in our study cohort (42%). Fifth, the surgical location of thromboembolic disease was judged intraoperatively and according to photographs of the pulmonary vascular specimens. Due to the inherent risks of the procedure, no full-thickness biopsies at different levels of the pulmonary arteries were performed.

## 6. Conclusions

Our results show that CTPA is highly sensitive to predicting the level of disease in CTEPH patients with a moderate interobserver agreement. The radiological assessment showed a trend for classifying more patients as proximal disease. This imaging-based classification is associated with the improvement of hemodynamics after pulmonary endarterectomy and may be used in the future to support a surgeons’ decision making to offer surgery to CTEPH patients.

## Figures and Tables

**Table 1:** Baseline demographics.

Female		18 (42%)
Age at diagnosis [years]		57 ± 16
Height [cm]		173 ± 9
Weight [m]		82 ± 18
NYHA classification at diagnosis	I	1 (2%)
	II	13 (30%)
	III	25 (58%)
	IV	4 (9%)
Smoking status	Current	7 (16%)
	Former	18 (42%)
	Never	18 (42%)
Pack years		31 ± 24
Pulmonary artery pressure—systolic [mmHg]		67 ± 16
Pulmonary artery pressure—mean [mmHg]		42 ± 10
Pulmonary vascular resistance [Wood Units]		6.4 ± 2.8
Cardiac Index [l/min]		2.6 [2.2, 3.2]
Right ventricular—right atrial (RV-RA) gradient [mmHg]		59 [45, 70]
Six-minute walking test—distance [m]		423 ± 129

Categorical data are given as count (percentage) and continuous data are given as the mean ± standard deviation or median [inter-quartile range].

**Table 2:** Comparison of radiological versus surgical classification of the level of disease in patients with chronic thromboembolic pulmonary hypertension.

**Radiological Consensus Classification** **(Level)**	**Surgical Classification (Level)**				
		1	2	3	4
	1	10	2	0	0
	2a	4	10	9	0
	2b	0	0	3	0
	3	0	0	4	1

**Table 3:** Baseline hemodynamic parameters according to CT classification of thromboembolic level of disease.

	Level of Disease Classification According to CT			
	1 (*n* = 12)	2a (*n* = 23)	2b/3 (*n* = 8)	*p*-Value
Age (years)	66 ± 11 *	54 ± 17	47 ± 11 *	0.018
Male sex (%)	9 (75%)	12 (52%)	4 (50%)	0.38
Six-minute walking test [m]	377 ± 117 *	412 ± 122	523 ± 128 *	0.034
Moderate or severe tricuspid regurgitation	2 (16%)	7 (30%)	2 (25%)	0.81
Tricuspid regurgitation pressure gradient [mmHg]	61 [42, 75]	59 [38, 70]	63 [47, 72]	0.86
LV ejection fraction [%]	61 ± 4	60 ± 7	62 ± 4	0.77
Systolic pulmonary artery pressure [mmHg]	72 ± 13	66 ± 16	65 ± 18	0.55
Mean pulmonary artery pressure [mmHg]	46 ± 9	41 ± 10	38 ± 8	0.22
Cardiac index [l/min]	2.4 [1.9, 2.7]	3.0 [2.3, 3.3]	2.6 [2.1, 2.9]	0.12
Pulmonary vascular resistance [Wood Units]	8.3 ± 3.3 *	5.4 ± 2.0 *	6.3 ± 2.7	0.045

Categorical data are given as count (percentage) and continuous data are given as the mean ± standard deviation or median [inter-quartile range]. * Indicates significant differences (*p* < 0.05) between subgroups after post hoc analysis using Bonferroni correction of the Kruskal–Wallis test. Abbreviations: LV, left ventricular; RA, right atrium; RV, right ventricle.

**Table 4:** Changes in hemodynamic parameters between preoperative baseline measurements and postoperative 3-months (six-minute walking test and echocardiography) or 12-months (right heart catheterization) follow-up.

	Thromboembolism Classification According to CT			
	1 (*n* = 12)	2a (*n* = 23)	2b and 3 (*n* = 8)	*p*-Value
Tricuspid regurgitation pressure gradient [mmHg]	31 ± 27	18 ± 24	25 ± 14	0.37
Systolic pulmonary artery pressure [mmHg]	35 ± 18	25 ± 19	15 ± 15	0.079
Mean pulmonary artery pressure [mmHg]	22 ± 12 ^a^	14 ± 11	8 ± 6 ^a^	0.029
Pulmonary vascular resistance [Wood Units]	5.3 ± 3.7 ^b,c^	2.1 ± 2.8 ^c^	1.6 ± 1.4 ^b^	0.011

Categorical data are given as count (percentage) and continuous data are given as the mean ± standard deviation or median [inter-quartile range]. ^a^ *p* = 0.029 with post hoc Bonferroni correction; ^b^ *p* = 0.036 with post hoc Bonferroni correction; ^c^ *p* = 0.018 with post hoc Bonferroni correction. Abbreviations: RA, right atrium; RV, right ventricle.

## Data Availability

Upon reasonable request to the corresponding author.

## Abbreviations

CT: computed tomography; CTEPH, chronic thromboembolic pulmonary hypertension; CTPA, computed tomography pulmonary angiography; ICC, intraclass correlation coefficient; PA, pulmonary artery; V/Q scintigraphy, ventilation/perfusion scintigraphy.
